# Acceptability of mobile-phone reminders for routine childhood vaccination appointments in Nigeria – a systematic review and meta-analysis

**DOI:** 10.1186/s12913-021-07296-1

**Published:** 2021-11-26

**Authors:** Paul Eze, Sergius Alex Agu, Ujunwa Justina Agu, Yubraj Acharya

**Affiliations:** 1 Department of Health Policy and Administration, Pennsylvania State University, University Park, PA 16802 USA; 2 Department of Paediatrics, University of Nigeria Teaching Hospital, Ituku-Ozalla, Enugu, Nigeria; 3 Department of Paediatrics, Enugu State University Teaching Hospital, Parklane, Enugu, Nigeria

**Keywords:** Mobile-phone reminders, Willingness to receive, Routine immunization, Systematic review, Meta-analysis, Nigeria

## Abstract

**Background:**

Mobile-phone reminders have gained traction among policymakers as a way to improve childhood vaccination coverage and timeliness. However, there is limited evidence on the acceptability of mobile-phone reminders among patients and caregivers. This systematic review and meta-analysis aimed to evaluate the ownership of mobile-phone device and the willingness to receive mobile-phone reminders among mothers/caregivers utilizing routine childhood immunization services in Nigeria.

**Method:**

MEDLINE, Scopus, CINAHL, CNKI, AJOL (African Journal Online), and Web of Science were systematically searched for studies on the acceptability of mobile-phone reminders for routine immunization appointments among mothers/caregivers in Nigeria. Studies were assessed for methodological quality using the Newcastle Ottawa Scale and JBI critical appraisal checklists. Meta-analysis was conducted using random-effects model to generate pooled estimates (proportion) of mothers who owned at least one mobile phone and proportion of mothers willing to receive mobile-phone reminders.

**Results:**

Sixteen studies (13 cross-sectional and three interventional) involving a total of 9923 mothers across 15 states and the Federal Capital Territory Abuja met inclusion criteria. Pooled estimates showed that the proportion of mothers who owned at least one mobile phone was 96.4% (95% CI = 94.1–98.2%; *I*^2^ = 96.3%) while the proportion of mothers willing to receive mobile-phone reminders was 86.0% (95% CI = 79.8–91.3%, *I*^2^ = 98.4%). Most mothers preferred to receive text message reminders at least 24 h before the routine immunization appointment day, and in the morning hours. Approximately 52.8% of the mothers preferred to receive reminders in English, the country’s official language.

**Conclusion:**

Current evidence suggests a high acceptability for mobile-phone reminder interventions to improve routine childhood immunization coverage and timeliness. Further studies, however, are needed to better understand unique regional preferences and assess the operational costs, long-term effects, and risks of this intervention.

**Systematic review protocol registration:**

PROSPERO CRD42021234183.

**Supplementary Information:**

The online version contains supplementary material available at 10.1186/s12913-021-07296-1.

## Introduction

Routine childhood immunization has been remarkably successful and cost-effective in preventing infectious diseases worldwide [1]. In addition to saving millions of lives, vaccination generates immense economic benefits to society and saves the society the costs of reacting to outbreaks [2–4]. Immunization is also central to the Sustainable Development Goals (SDG) [4]. Like many other health care services that require repeated visits to the health facility due to timed scheduling of care, routine childhood immunization is characterized by poor compliance and attrition [5]. Inadequate information about immunization schedules and service arrangements with difficult-to-remember childhood vaccine series (with multiple appointments at various ages) is a key factor contributing to caregivers defaulting from routine immunization [6, 7].

Reminder systems, which work through a variety of mechanisms, including phone calls, letters, postcards, and email, are meant to prompt the patient [8], and have been shown to improve immunization compliance and timeliness [9, 10]. Among the various types of reminder systems, mobile phone reminders have been found to be the most effective [9, 11]. Hence, an increasing number of studies recommend policymakers and operations managers to consider integrating mobile-phone reminders into routine immunization programmes [9, 11, 12]. A number of systematic reviews have synthesized the evidence on the effectiveness of mobile phone reminders on the uptake of immunization in low- and middle-income countries (LMICs) [13–16]. While all of these reviews suggest that phone reminders are generally effective, these exists a gap in our understanding of a key mechanism: acceptability. For mobile-phone reminders to encourage action by the recipient (in this case, take the child to the health facility for immunization), the reminders first need to be acceptable [17].

Against this background, this study aims to systematically review the literature to: (a) evaluate mobile-phone ownership among caregivers utilizing the routine vaccination service in Nigeria, (b) assess caregivers’ willingness to receive mobile-phone reminders for routine childhood vaccination appointments, and (c) assess mobile-phone reminder preferences in terms of timing, frequency, and mode. We purposefully limit the analysis to studies conducted in Nigeria in an effort to inform the country’s policy efforts on immunization. Despite efforts by the Nigerian government and international partners to ensure optimal utilization of routine immunization, over four million children in Nigeria still miss out on vaccinations every year [18]. Nigeria is one of 10 countries that account for more than 60% of the 19.7 million children that did not receive complete doses of Diphtheria-Pertussis-Tetanus (DPT) vaccine in 2019 [19, 20]. Nigeria is one of only ten countries in the world with measles vaccine coverage of less than 50% [21, 22]. Nearly 40% of under-five mortality in Nigeria, accounting for about 15% of global child deaths, have been attributed to vaccine preventable diseases [23].

Studies across Nigeria have demonstrated that lack of accurate information about immunization and immunization services is significantly associated with incomplete immunization [24–28]. Forgetting vaccination appointments is widespread among mothers/caregivers [26, 27]. The recent explosion in mobile phone ownership offers a promising opportunity to leverage on this proven mobile health (mHealth) interventions to improve vaccination rates [29, 30]. However, like in the case of LMICs generally, several studies in Nigeria have demonstrated the utility of mobile-phone reminders in improving routine immunization coverage and timeliness [31–33], with scant evidence on caregivers’ *willingness* to receive mobile phone reminders for routine childhood immunization appointments.

## Methods

The protocol for this systematic review was published on PROSPERO, with registration number CRD42021234183, and the review findings were reported according to the MOOSE and PRISMA guidelines [34, 35].

### Search strategy and study selection

We searched MEDLINE via PubMed, Scopus: https://www.scopus.com/search/form.uri?display=basic#basic, Cochrane Database of Systematic Reviews and Cochrane Central Register of Controlled Trials (Cochrane Library, Wiley), CINAHL (Cumulative Index to Nursing and Allied Health Literature) via EBSCOHost, PsycINFO via ProQuest, CNKI (China National Knowledge Infrastructure), AJOL (African Journal Online): https://www.ajol.info/index.php/ajol/search, Science Citation Index Expanded, Social Sciences Citation Index and Arts & Humanities Citation Index (Web of Science Core Collection) for published studies of any design that reported mothers’ or caregivers’ willingness to receive mobile-phone reminders in Nigeria from 01 January 2001 to 31 December 2020. The search time span starting from 2001 was chosen as pertinent to the year mobile phone services were introduced in Nigeria in 2001 [30]. Search was conducted over 4 weeks in January and February 2021. We used search terms covering mobile-phone reminders, phone call reminders, short message services (SMS) reminders (SMS, texts, text message), routine immunization, willingness to receive, acceptability and Nigeria (Supplement 1 - Search strategy). We also searched Google Scholar: https://scholar.google.com/; Semantic Scholar: https://semanticscholar.org/; SCIELO (Scientific Electronic Library Online): https://scielo.org/en/; and websites of the National Postgraduate Medical College of Nigeria for eligible studies: https://npmcn.edu.ng/; United Nation Children Fund (UNICEF), Nigeria: https://unicef.org/nigeria/; and World Health Organization (WHO), Nigeria: https://afro.who.int/countries/nigeria. We also searched Grey literature websites of the NYAM (New York Academy of Medicine) Grey: https://catalog.nyam.org/; and Open Grey: http://opengrey.eu/. Finally, we also sought for relevant articles from the references of studies identified through the database search. We only considered studies published in English as scientific/research articles in Nigeria are reported in English. The authors of relevant papers were contacted for missing information.

The search was independently conducted by two authors (PE and SAA); both authors search independently across all databases. Identified studies were pooled into Mendeley® Reference Manager and duplicates were identified and excluded. After undergoing a moderation exercise to ensure uniform application of inclusion criteria, the two authors independently assessed the titles and abstracts for eligibility applying the inclusion criteria. Discrepancies were resolved by discussion. Finally, full text of each remaining articles was assessed against the inclusion criteria.

### Eligibility criteria

Peer-reviewed studies of any design; published and unpublished, were included in the review if they were conducted among adults aged 18 years or more, residing in Nigeria, and reported the proportion of mothers/caregivers who owned a mobile phone or proportion of mothers/caregivers willing to receive mobile phone reminder (SMS or phone calls), or enough data to compute these estimates. We excluded studies that reported willingness to receive mobile phone reminders for hospital appointments, medication adherence, health behavioural change, and/or vaccination in adults (such as human papillomavirus (HPV) vaccine, tetanus vaccine, etc.). We also excluded case series, reviews, commentaries, letters, and editorials.

### Data extraction

Two authors (PE and SAA) extracted data from the included studies including authors, year of study, study design, location/region, study setting (rural or urban), description of study population, sample size (number of subjects involved), mean age with standard deviation, proportion of study participants with minimum of secondary education, ownership of a mobile phone, and number of mothers/caregivers willing to receive mobile-phone reminders. Information on mothers’/caregivers’ mobile-phone reminder preferences (SMS or phone calls), preferred frequency, and ideal timing of mobile phone reminders were also collected. Microsoft Excel© was used to organize extracted data. Disagreements were resolved through discussion until there was 100% agreement.

### Quality assessment

Two reviewers (PE and UJA) independently assessed methodological quality to establish the internal validity and risk of bias of included studies using the modified Newcastle Ottawa Scale for cross-sectional studies [36], and the Joana Briggs Institute (JBI)‘s critical appraisal checklists for interventional studies [37]. Studies were rated as ‘high quality’ or ‘medium quality’ if they scored 7–8 points or 5–6 points, respectively. Otherwise, the study was rated as ‘low quality’. Inter-rater discrepancies were resolved by discussion until 100% agreement reached.

### Data synthesis

Data analysis was performed according to the guidelines specified in the Joanna Briggs institute (JBI)‘s Manual for Evidence Synthesis [38, 39]. Descriptive statistics and narrative synthesis were used to summarize the characteristics of included studies. Prevalence and 95% confidence interval (CI) for ownership of a mobile-phone device and willingness to receive mobile-phone reminders were estimated for each included study. Confidence intervals were estimated using the one-sample exact binomial (Clopper-Pearson) procedures [40]. Pairwise meta-analysis using the random-effects (DerSimonian-Laird) model were performed to pool individual results using the *Metaprop* Stata command with Freeman-Tukey double arcsine transformation (FTT) [40, 41]. Analyses were conducted using Stata version 16.1 (STATA Corp, College Station, TX). An α (alpha) of 0.5 was uses as the cutoff for statistical significance.

Sensitivity analyses were first performed for the influence of studies with sample size less than 400 participants – as studies with small sample size are more likely to exaggerate study outcomes [37]. Pooled estimates were also assessed for influence of studies with sample size outliers. Lastly, pooled estimates were also re-assessed after excluding interventional studies. Subgroup analyses were performed for the periods the studies were published (2011–2015, and 2016–2020), region of study (northern regions vs southern regions), study setting (urban, mixed, and rural), and proportion of study participants with at least secondary school education (< 90% and ≥ 90%). Meta-regression analyses were performed to assess the impact of modifier variables: study period, study region, study setting, and proportion of mothers/caregivers with at least secondary education (continuous variable), on the meta-estimate proportion. Finally, evidence of publication bias was assessed by examining the symmetry of the funnel plot using sample size as the measure of accuracy [42], performing Egger’s test for funnel-plot asymmetry, and using the trim-and-fill method.

### Assessment of quality of evidence

The overall quality of evidence was assessed using GRADE (Grading of Recommendations, Assessment, Development and Evaluation) for the meta-analysis pooling data from all included studies [43]. Scoring of evidence started at high-quality evidence which was downgraded one level if one of the following prespecified criteria was present: (1) poor methodological quality (downgraded if ≥25% of the studies included in the meta-analysis used inappropriate sampling method or statistical analyses; (2) imprecision (downgraded if ≥25% of the included studies did not present minimum required sample size); (3) indirectness (downgraded if ≥25% of the included studies did not use valid and reliable methods for data collection, such as validated questionnaires that had been trialed, piloted, or published previously) and (4) inconsistency (downgraded if the confidence interval was wider than or equal to 5%). These pre-specified criteria were defined considering the items of Joana Briggs that correspond to the GRADE system criteria [38, 39].

## Results

### Selection of studies

**Fig. 1:**
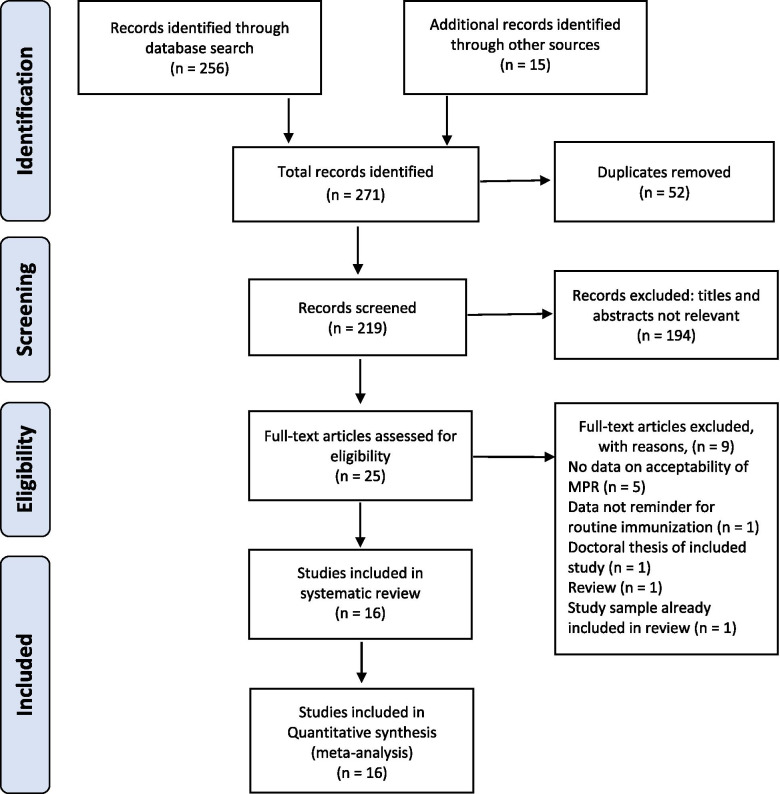
PRISMA Flow Diagram**.** PRISMA: Preferred Reporting Items for Systematic Reviews and Meta-Analyses

The study selection process is illustrated in a PRISMA flow diagram (Fig. 1). The databases searches returned 256 studies, and 15 additional studies were identified through Google Scholar, Semantic Scholar, and handsearching reference lists of relevant studies. After duplicates were removed, 219 studies were screened for relevance. On applying the selection criteria, 194 studies were excluded. Hence, 25 studies full texts articles were assessed and further screened using the predesigned selection criteria. Sixteen studies met the inclusion criteria for data extraction and were included in the review [44–59], while nine studies were excluded for the following reasons: study did not report data on acceptability of mobile-phone reminders (*n* = 5) [32, 60–63], reported data was not specific for routine immunization appointments (*n* = 1) [64], review (n = 1) [65], doctoral thesis of an article already included in the review (*n* = 1) [66], study reported data from a sample already included in review (n = 1) [67].

### Description of included studies

**Fig. 2:**
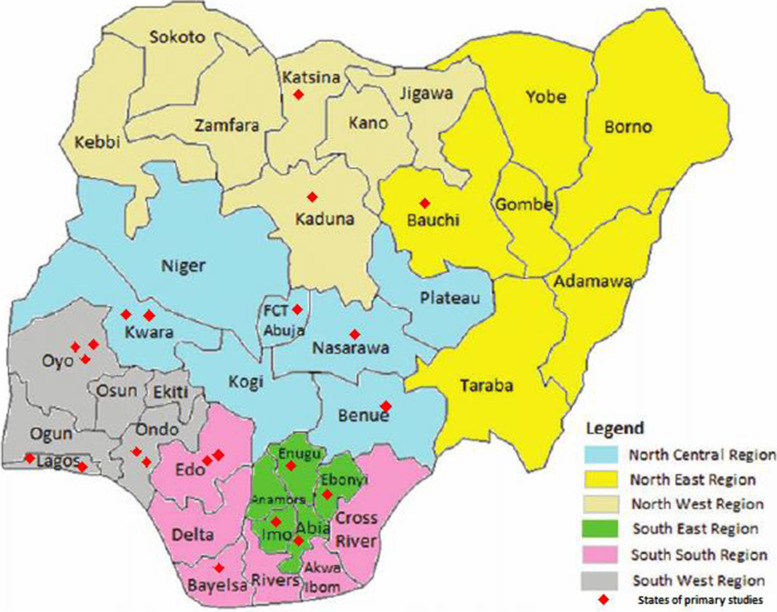
Map of Nigeria showing the states where included primary studies were conducted. Source: Original map was culled from Gayawan E, Arogundade ED, Adebayo SB. Possible determinants and spatial patterns of anaemia among young children in Nigeria: a Bayesian semi-parametric modelling. Int Health. 2014;6(1):35–45

Sixteen peer-reviewed primary studies, presented in Table 1, were included in this review: 13 cross-sectional studies and three interventional studies (one non-randomized controlled trial and two randomized controlled trial) compromising a total of 9923 participants (Mean age = 28.15 years, SD = 4.59 years, 99.8% mothers, 95.3% married/co-habiting) across 15 states and the Federal Capital Territory (FCT), Abuja (Fig. 2). Primary studies were published between 2012 to 2020, and were undertaken in all six geopolitical zones of the country: South-west (*n* = 7) [44, 45, 48, 52, 54–56], South-east (n = 5) [47, 48, 51, 58], North-central (*n* = 4) [46, 48, 53, 59], South-south (*n* = 3) [48, 50, 57], North-west (*n* = 2) [48, 49], North-east (n = 1) [48], and the FCT Abuja (n = 1) [48]. Thirteen studies were conducted in urban settings, two in rural [48, 56], and one in a mixed peri-urban setting [45].

**Table 1:** Summary of the descriptive characteristics of included studies

Author,	Study design	Study location (Region, State, and Setting)	Study population	Sample size (N)	Mean Age (SD) (yrs)	Mobile-phone ownership, n (%)	Willingness to receive MPR, n (%)	Study Quality
Ajayi et al., 2015 [44]	Cross-sectional	Region: SW State: Lagos Setting: Urban	Mothers at home in Mushin LGA, Lagos	400	28.1 (9.50)	374 (93.5%)	306 (76.5%)	High
Akinrinde et al., 2018 [45]	Cross-sectional	Region: SW State: Ondo Setting: Mixed (Rural, peri-urban, and urban)	Mothers of infants attending routine immunization clinics in 24 randomly selected PHC centers	615	28.5 (6.01)	529 (86.0%)	613 (99.7%)	High
Balogun et al., 2012 [52]	Cross-sectional	Region: SW State: Lagos Setting: Urban	Mothers attending the Lagos University Teaching Hospital (LUTH)‘s child welfare clinic, Lagos	399	31.1 (4.70)	391 (98.0%)	308 (77.2%)	High
Bello et al., 2020 [53]	Cross-sectional	Region: NC State: Nasarawa Setting: Urban	Mothers of children in the Medical Ward of Dalhatu Araf Specialist Hospital (DASH), Lafia	135	27.5 (5.82)	124 (91.9%)	111 (82.2%)	Low
Brown & Oluwatosin, 2017 [54]	Interventional	Region: SW State: Oyo Setting: Urban	Mothers of infants attending routine immunization clinics in four randomly selected communities’ PHC centers in Ibadan	595	27.5 (5.82)	590 (99.2%)	584 (98.2%)	High
Brown et al., 2015 [55]	Cross-sectional	Region: SW State: Oyo Setting: Urban	Mothers of infants attending routine immunization clinics in four randomly selected communities’ PHC centers in Ibadan	614	29.0 (4.90)	607 (98.8%)	584 (95.1%)	High
Dipeolu 2017 [56]	Cross-sectional	Region: SW State: Oyo Setting: Rural	Mothers of Infants attending Routine immunization clinics in two randomly selected rural LGAs	366	26.1 (5.55)	366 (100.0%)	340 (92.9%)	High
Eze & Adeleye, 2015 [57]	Interventional	Region: SS State: Edo Setting: Urban	Caregivers of infants attending routine immunization from eight randomly health facilities in Egor LGA in Edo State	905	30.5 (5.43)	896 (99.0%)	843 (93.1%)	High
Eze et al., 2018 [58]	Interventional	Region: SE State: Ebonyi Setting: Urban	Caregivers of infants accessing immunization services in rural health facilities in Abakaliki.	290	27.0 (5.21)	290 (100.0%)	285 (98.3%)	Moderate
Ibraheem & Akintola, 2017 [59]	Cross-sectional	Region: NC State: Kwara Setting: Urban	Mothers/caregivers bringing their newborns for their first set of vaccines at two public hospitals in Ilorin West LGA	526	28.5 (4.80)	488 (92.8%)	363 (69.0%)	High
Ibraheem et al., 2018 [46]	Cross-sectional	Region: NC State: Kwara Setting: Urban	Caregivers bringing their newborns for their first set of vaccines at two public hospitals in Ilorin West LGA	536	28.5 (4.80)	526 (98.1%)	363 (67.7%)	High
Odinaka et al., 2018 [47]	Cross-sectional	Region: SE State: Imo Setting: Urban	Mothers of infants attending the immunization clinic of Federal Medical Centre Owerri, Imo State	253	30.4 (7.10)	244 (96.4%)	156 (61.7%)	Moderate
Oladepo et al., 2019 [48]	Cross-sectional	Region: All six regions States: Abia, Bauchi, Benue, Bayelsa, Katsina, Ondo, and FCT Setting: Rural	Mothers of infants attending immunization clinics in seven randomly selected states	3500	26.7 (5.50)	3440 (98.3%)	3113 (88.9%)	High
Onoja-Alexander et al., 2018 [49]	Cross-sectional	Region: NW State: Kaduna Setting: Urban	Randomly selected caregivers attending routine childhood immunization clinic in ABUTH Shika, Zaria	300	28.7 (4.22)	235 (78.3%)	251 (83.7%)	High
Sadoh & Okungbowa, 2014 [50]	Cross-sectional	Region: SS State: Edo Setting: Urban	A conveniently selected sample of mothers of infants attending immunization clinic at the Institute of Child Health, University of Benin, Benin City.	203	30.5 (5.07)	188 (92.6%)	127 (62.6%)	Low
Tagbo et al., 2020 [51]	Cross-sectional	Region: SE State: Enugu Setting: Urban	Caregivers bringing their infants for immunization in five randomly selected health facilities in Enugu metropolis	286	29.6 (6.97)	278 (97.2%)	255 (89.2%)	Moderate

ABBREVIATIONS: *MPR* Mobile-phone reminders, *SD* Standard deviation, *NC* North-Central, *NE* North-East, *NW* North-West, *SE* South-East, *SS* South-South, *SW* South-West, *FCT* Federal Capital Territory, Abuja. *ABUTH* Ahmadu Bello University Teaching Hospital

Of the 16 included primary studies, 11 studies (69%) were rated as high quality, three studies (19%) rated as moderate quality, and two studies (12%) which used convenience sampling were rated as low quality. Of note, however, is that probability random sampling was used to identify participants in 14 of the 16 included primary studies with total sample size of 9585 participants (96.6% of the overall study sample). Hence, sampling methods employed in primary studies ensures that results obtained from included study participants approximates results from the entire population. However, two studies with total sample size (338, or 3.4%) employed the convenience sampling method [50, 53].

### Ownership of mobile phone

**Fig. 3:**
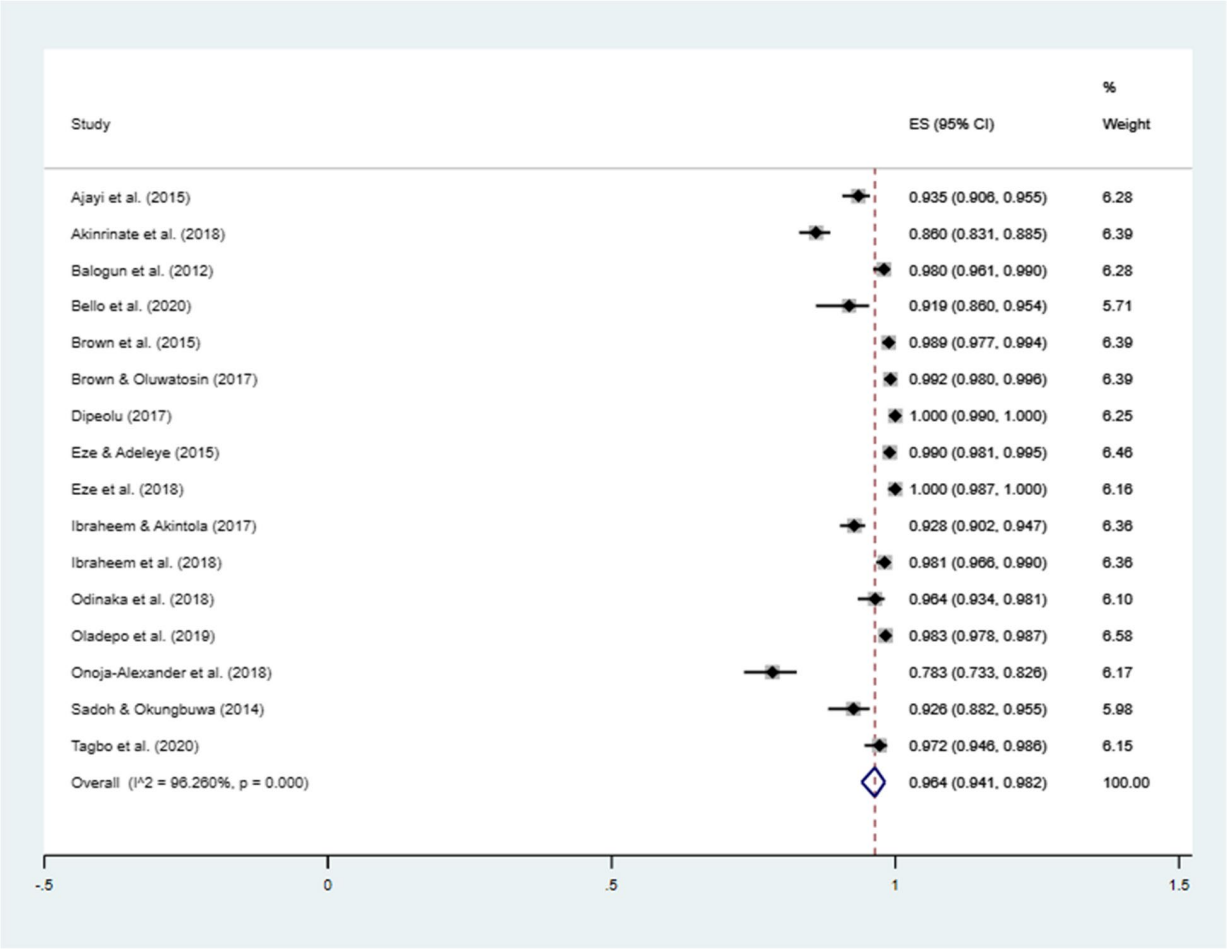
Forest plot showing proportion of mothers that own at least one mobile-phone device

Meta-analysis of pooled data from included primary studies shows that proportion of mothers who owned at least one mobile phone was 96.4% (95% CI = 94.1–98.2%; *I*^2^ = 96.3%) – Fig. 3. The lowest proportion (78.3%) of mobile-phone ownership among mothers was in Kaduna State, North-west region in 2018 [49] while the highest proportion (100.0%) was reported in Ebonyi State, South-east region in 2018 [67], and Oyo State, South-west in 2017 [56]. About 5.0% of respondents owned more than one phone [56] while about a quarter of mothers has more than one active lines [48, 56]. Sensitivity analysis showed that pooled estimate was not affected by studies with sample size less than 400 mothers (pooled proportion = 96.6%; 95% CI = 93.8–98.6%; *I*^2^ = 96.6%), study with sample size outlier [48] (pooled proportion = 96.2%; 95% CI = 93.4–98.3%; *I*^2^ = 96.3%), nor by studies with an interventional design (pooled proportion = 95.3%; 95% CI = 92.2–97.6%; *I*^2^ = 96.5%).

Sub-group analysis showed that ownership of mobile-phones was about 6.0% higher among mothers in the Southern region than among mothers in the Northern region – Table 2. However, there was no substantial difference in mobile-phone ownership among mothers based on study publication period, setting, design, and quality of included primary studies. Meta-regression analysis demonstrated that a one-percent increase in the proportion of mothers with at least secondary school education (continuous variable) was associated with a 0.398 unit increase in mobile-phone ownership (*p* = 0.022). However, study publication period, geopolitical region, and study setting were not statistically significant modifiers.

**Table 2:** Ownership of mobile-phone by various study characteristics

Ownership of mobile phones	No. of studies	Study sample	Pooled estimate	95% CI	***I***^**2**^
**Study period**					
° 2011 to 2015	5	2521	0.970	0.943–0.989	91.1%
° 2016 to 2020	11	7402	0.961	0.926–0.985	97.2%
**Region**					
° North (West and Central)	4	1497	0.915	0.817–0.987	96.8%
° South (West, South, and East)	11	4926	0.975	0.949–0.992	95.6%
° Multi-regions **	1	3500	0.983	0.978–0.987	–
**Study setting**					
° Rural settings	2	3866	0.986	0.982–0.990	–
° Mixed (rural and urban)	1	615	0.860	0.831–0.885	–
° Urban settings	13	5442	0.963	0.937–0.983	94.9%
**Educational status**					
° Secondary education, < 90%	8	3177	0.939	0.875–0.981	97.3%
° Secondary education, ≥ 90%	8	6746	0.982	0.969–0.991	84.4%
**Study design**					
° Cross-sectional	13	8133	0.953	0.922–0.976	96.5%
° Interventional studies	3	1790	0.994	0.987–0.999	0.0%
**Quality of included studies**					
° High quality	11	8756	0.963	0.934–0.985	97.2%
° Low and Medium quality	5	1167	0.966	0.923–0.992	90.4%

** Includes states in all regions

NOTE: The pooled estimates are the proportions of respondents who owned a mobile phone. For example, in the 5 studies conducted between 2011 and 2015 and included in this review, 97% of respondents owned a mobile phone

### Acceptability of mobile-phone reminders

**Fig. 4:**
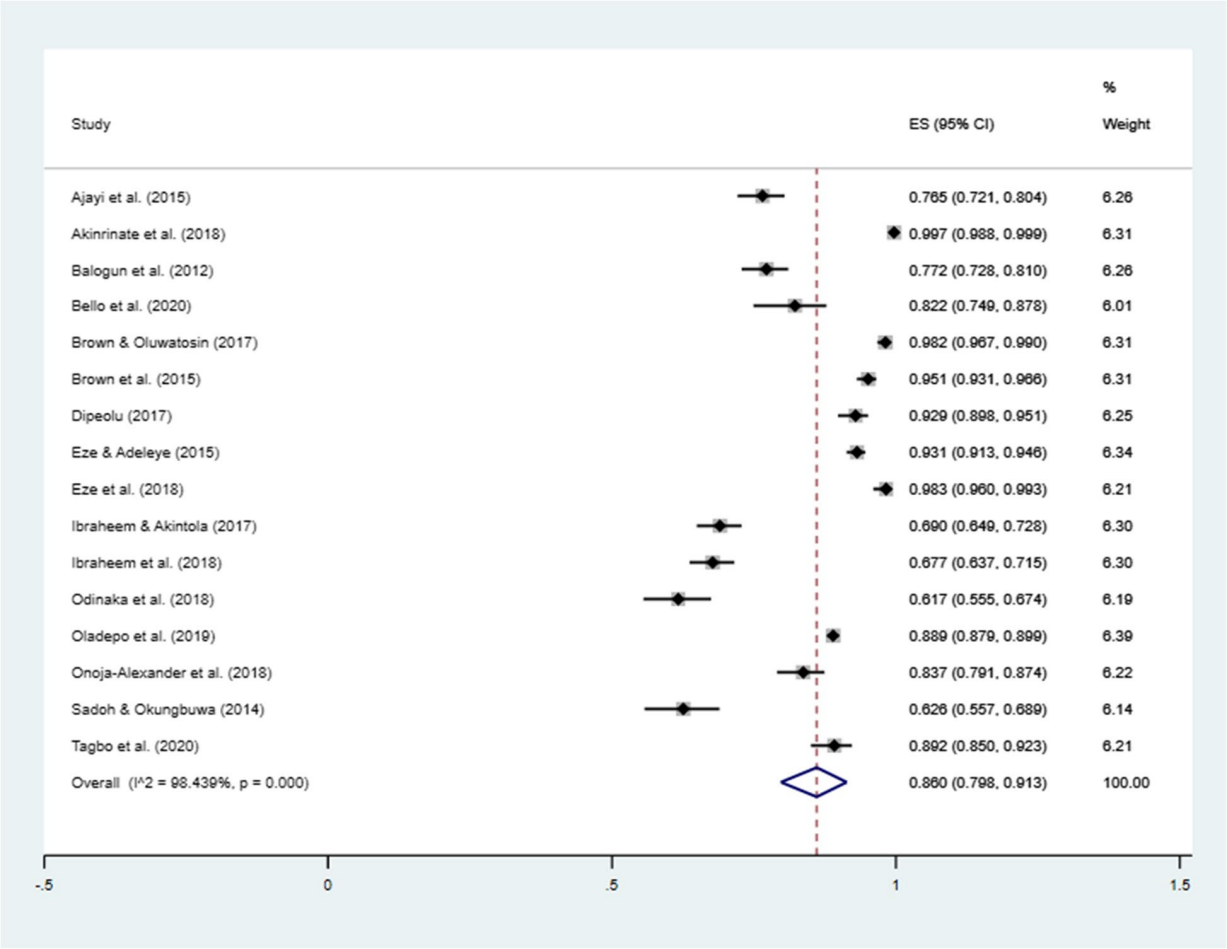
Forest plot showing proportion of mothers willing to receive mobile-phone reminders

Pooled estimate for acceptability of (willing to receive) mobile-phone reminders was 86.0% (95% CI = 79.8–91.3%, *I*^2^ = 98.4%) – Fig. 4. Acceptability of mobile-phone reminders is lowest (61.7%) in Imo State, South-east region in 2018 [47] and highest in Ondo State, South-west region in 2018 [45]. In sensitivity analyses, the pooled estimate did not change substantially from the overall results when studies with sample size less than 400 mothers were excluded (pooled proportion = 88.8%; 95% CI = 80.4–95.1%; *I*^2^ = 98.9%) nor when studies with sample size outlier were excluded [48] (pooled proportion = 85.8%; 95% CI = 78.0–92.2%; *I*^2^ = 98.5%). However there the acceptability slightly decreased when interventional studies were excluded (pooled proportion = 82.6%; 95% CI = 74.9–89.1%; *I*^2^ = 98.4%).

Sub-groups analysis showed slight variations in the acceptance of mobile-phone reminders based the survey period, geopolitical region, setting, and educational studies of participants. Acceptability of mobile-phone reminders was slighter higher in the period 2016–2020 compared with the earlier period (2011–2015), higher among mothers in the southern regions than mothers in the northern regions, higher among mothers in the rural settings than mothers in the urban settings (Table 3). Meta-regression analysis demonstrated that the proportion of mothers with at least secondary school education (continuous variable), study period, geopolitical region and study setting were not statistically significant modifiers of willingness to receive mobile-phone reminders.

**Table 3:** Acceptability of mobile-phone reminders by various study characteristics

Acceptability of mobile-phone reminders	No. of studies	Study sample	Pooled estimate	95% CI	***I***^**2**^
**Study period**					
° 2011 to 2015	5	2521	0.828	0.703–0.924	98.1%
° 2016 to 2020	11	7402	0.874	0.793–0.938	98.7%
**Region**					
° North (West, Central, and East)	4	1497	0.757	0.672–0.833	92.0%
° South (West, South, and East)	11	4926	0.889	0.809–0.949	98.4%
° Multi-regional	1	3500	0.889	0.879–0.899	–
**Study setting**					
° Rural settings	2	3866	0.894	0.884–0.903	–
° Mixed (rural and urban)	1	615	0.997	0.988–0.999	–
° Urban settings	13	5442	0.833	0.749–0.903	98.3%
**Educational status**					
° Secondary education, < 90%	8	3177	0.842	0.722–0.933	98.5%
° Secondary education, ≥ 90%	8	6746	0.877	0.794–0.941	98.6%
**Study design**					
° Cross-sectional	13	8133	0.826	0.749–0.891	98.4%
° Interventional studies	3	1790	0.968	0.927–0.993	0.0%
**Quality of included studies**					
° High quality	11	8756	0.879	0.811–0.934	98.6%
° Low and Medium quality	5	1167	0.814	0.628–0.946	98.1%

** Includes states in all regions

NOTE: The pooled estimates are the proportions of respondents who were willing to receive mobile-phone reminders. For example, in the 5 studies conducted between 2011 and 2015 and included in this review, 82.8% of respondents were willing to receive mobile-phone reminders for routine childhood vaccination appointments

### Mothers’ mobile-phone reminder preferences

Meta-analysis of nine primary studies [45–47, 49–53, 55] including 2865 mothers who indicated willingness to receive mobile-phone reminders showed that 57.0% (95% CI = 39.7–73.5%; *I*^2^ = 98.8%) of participants preferred to receive phone-call reminders while about two-thirds (pooled proportion = 63.5%; 95% CI = 48.7–77.1%; *I*^2^ = 98.4%) of participants preferred to receive text (SMS) message reminders. However, two primary studies reported differed data on mothers willing to accept both modes of reminders: while Akinrinde and colleagues reported about a quarter of mothers (28.3%) are willing to receive both types of reminders [45], Bello and colleagues reported a negligible proportion (2.2%) of mothers prefer both modes of reminders [53]. Mothers who had post-secondary education were more likely to prefer text messages [45, 46, 50].

A majority of mothers preferred to receive mobile-phone reminders for routine childhood immunization reminders at least 24 h before the appointment day [45, 47, 50–52], and to receive these reminders in the morning hours [45, 48, 56]. However, a significant proportion of mothers reported willingness to receive these reminders at any hours of the day [45, 48, 56], although mothers in these studies were asked what time/hours they were willing to receive text (SMS) message reminders, not phone-call reminders. Lastly, meta-analysis of six primary studies [45, 46, 48, 52, 56, 59] including 5126 mothers who indicated willingness to receive mobile phone reminders showed that a slim majority of mothers (pooled proportion = 52.8%; 95% CI = 34.5–70.7%; *I*^2^ = 99.2%) preferred to receive mobile-phone reminders in English language.

### Assessment of publication bias

Graphical assessment of the funnel plot suggests absence of publication bias (Supplement 2). Objective assessment of publication bias using the Egger test also indicated absence of evidence of publication bias (*p*-value = 0.1450) (Supplement 3). Trim-and-fill method for assess publication bias did not demonstrate any evidence of publication bias (Supplement 4).

### Quality of evidence

The quality of evidence for the ownership of mobile phones among mothers using the routine childhood vaccination services in Nigeria was graded as high – Table 4. There was no serious risk of bias, imprecision, indirectness, or inconsistency based on the pre-specified criteria. However, the quality of the evidence for acceptability of mobile-phone reminders and the mothers’ preferences for each mode of reminder (i.e., phone call reminders over SMS/text reminders and SMS/text reminders over phone call reminders) were graded as moderate based on serious inconsistency across included studies (Table 4).

**Table 4:** GRADE evidence table for Study’s outcome measures

Outcomes	Risk of bias^**a**^	Imprecision^**b**^	Indirectness^**c**^	Inconsistency^**d**^	Sample size	Quality
Ownership of mobile-phone	Not serious	Not serious	Not serious	Not serious	9923	High quality
Acceptability of mobile-phone reminders	Not serious	Not serious	Not serious	Serious	9923	Moderate quality
Preference: Phone calls over SMS/texts	Not serious	Not serious	Not serious	Serious	2865	Moderate quality
Preference: SMS/texts over phone calls	Not serious	Not serious	Not serious	Serious	2865	Moderate quality

^a^More than 25% of studies with a risk of bias (i.e., inappropriate sampling method or statistical analyses)

^b^More than 25% of studies with small sample size

^**c**^More than 25% of studies did not use valid and reliable methods for data collection

^d^Heterogeneity across the studies (confidence interval ≥ 5.0% between upper and lower limits)

## Discussion

This systematic review, to the best of our knowledge, presents the best available evidence on the ownership of mobile phones among Nigerian mothers/caregivers utilizing the routine childhood vaccine delivery service and their willingness to receive mobile-phone reminders. Our findings demonstrate that almost all mothers/caregivers utilizing the infant vaccine delivery system own at least one mobile phone. Although there are slight differences in mobile-phone ownership between mothers/caregivers in the Northern region and mothers/caregivers in the Southern region, there was no significant difference in the ownership over time and across different settings/communities. Our findings differ from an earlier study, 6 years ago, that reported only about half of women of reproductive age have access to mobile phones [68]. First, this could be due oversampling of women from urban settings in the northern region (Kano State, Jigawa State, and Kaduna State) – a region where we found slightly reduced mobile phone ownership than the rest of the country. Secondly, women in our study were mostly married/co-habiting (95.6%) – mobile phone ownership is significantly higher among married women who often get the phones as gifts from their spouses [29]. Our findings—the near-universal ownership of mobile phones—suggest that the adverse impact of the use of mobile technology on health care delivery on health disparities might be less concerning than previous studies have documented [5, 29, 30, 69]. Indeed, a number of prior studies have argued that the use of mobile phones in health care delivery can improve access, transparency, and equity of health services delivery in Nigeria [70, 71].

We found that a high proportion of mothers are willing and happy to receive mobile-phone reminders for their children’ routine immunization appointments. As already noted, health care services that require repeated visits to the health facility due to timed scheduling of care – including routine childhood immunization – are faced with the challenges of poor compliance and attrition [5]. These findings imply that the rapidly increasing mobile phone ownership in the country can be leveraged to deliver timely, and often educative, reminder messages to improve compliance and timeliness of immunization. We also found that the willingness to receive these reminders is increasing over time, which may be due to higher use of text messages in everyday life, including socially and in banking.

Additionally, our study also highlights some nuances for implementing mobile-phone reminders for routine childhood immunization in Nigeria. Although there were a few ‘universal’ preferences such as receiving mobile-phone reminders at least 24 h before the appointment day, most preferences such as choice over phone-call reminders vs SMS reminders or choice of language (English language or local language) were not. A statistically significant proportion of mothers who preferred phone-call reminders preferred a reminder in their local language, whereas statistically significant proportion of mothers who preferred text messages wanted the reminder in English language [39]. Geographically, one region where the use of text messages may not reach to the desired population is the northern region – a region with historically low immunization coverage [72] – where mothers are less inclined to receive mobile-phone reminders than the rest of the country. This could be due to factors such as the influence of religion and misperceptions of routine immunization [72] for which more engaged interventions than mobile-phone reminders alone will be needed. For designing such interventions, further studies are needed to better understand the influence of religion and culture on both health care utilization and adoption of technology [72].

### Strengths and limitations

With the number of studies (*n* = 16) and the population covered (*n* = 9923), this review represents, to the best of our knowledge, the most comprehensive and representative study on the acceptability of mobile-phone reminders for routine childhood immunization in Nigeria. We acknowledge that pooling prevalence rates from a range of studies conducted over a 10-year period (2011–2020) could affect reliability of our overall estimates; however, this approach enabled us to understand the trend in mobile phone ownership and acceptance of mobile-phone reminders over this period. Our pooled estimates should be considered with the high heterogeneity reported–a likely result of diverse population characteristics, particularly differences in religion, tribe/culture, and socio-economic status. Lastly, except for a single multi-region study [48], we could only retrieve studies from five of the six geopolitical zones in Nigeria, with no studies from the North-east region which covers six states and accounts for about 13.6% of Nigeria’s population [73]. This represents a critical gap in understanding the feasibility of successfully implementing this cost-effective m-health intervention in the region.

### Implication for practice and research

There appears to be broad acceptance of mobile-phone reminder interventions among mothers utilizing routine childhood vaccine delivery system in Nigeria. Given this widespread acceptance and its demonstrated cost-effectiveness and proven impact, health policymakers and stakeholders should consider including mobile-phone reminders as part of a multi-strategy approach to address slow adherence to routine immunization in the country. However, further studies, preferably utilizing qualitative design, are needed to explore and identify the factors (including religion and culture) why small, but nevertheless significant, proportion of mothers are unwilling to accept text reminders on health messages.

## Conclusion

Our findings demonstrate strong evidence for a high ownership of mobile phone devices among mothers utilizing the routine childhood delivery service in Nigeria, and also shows that most mothers are willing to receive mobile phone reminders for routine vaccination appointments. While these findings are encouraging, further studies are needed to better understand factors why some mothers decline to receive these reminders and appreciate nuanced regional and cultural differences in mothers’ preferences for mobile-phone reminder.

## Supplementary Information

**Additional file 1: Supplement 1.** Search strategy

**Additional file 2:** **Supplement 2.** Funnel plot for graphic assessment of publication bias

**Additional file 3:** **Supplement 3.** Egger test for objective assessment for evidence of publication bias

**Additional file 4:** **Supplement 4.** Trim-and-fill method for estimating potentially missing studies due to publication bias

## Data Availability

All data generated or analyzed during this study are included in this published article [and its supplementary information files].

## Abbreviations

AJOL: African Journal Online
CINAHL: Cumulative Index to Nursing and Allied Health Literature
CNKI: China National Knowledge infrastructure
FCT: Federal Capital Territory
FTT: Freeman-Tukey double arcsine transformation
GRADE: Grading of Recommendations, Assessment, Development and Evaluation
HPV: Human papillomavirus
JBI: Joana Briggs Institute
MOOSE: Meta-Analysis of Observational Studies in Epidemiology
PRISMA: Preferred Reporting Items for Systematic Reviews and Meta-Analyses
SCIELO: Scientific Electronic Library Online
SMS: Short Message Service

## References

[1] World Health Organization. Vaccines and immunization [Internet]. Health topics. 2021 [cited 2021 Jan 22]. Available from: https://www.who.int/health-topics/vaccines-and-immunization#tab=tab_1

[2] Piot P, Larson HJ, O’Brien KL, N’kengasong J, Ng E, Sow S (2019). Immunization: vital progress, unfinished agenda. Nature..

[3] Orensteina WA, Ahmed R (2017). Simply put: vaccination saves lives. Proc Natl Acad Sci.

[4] Feikin DR, Flannery B, Hamel MJ, Stack M, Hansen PM (2016). Vaccines for children in low- and middle-income countries. In: black RE, Laxminarayan R, Temmerman M, Walker N, editors. Reproductive, maternal, newborn, and child health: disease control priorities. Third edit.

[5] McLean S, Gee M, Booth A, Salway S, Nancarrow S, Cobb M, et al. Targeting the Use of Reminders and Notifications for Uptake by Populations (TURNUP): a systematic review and evidence synthesis. Heal Serv Deliv Res. 2014;2(34). 25642537

[6] Zewdie A, Letebo M, Mekonnen T. Reasons for defaulting from childhood immunization program: A qualitative study from Hadiya zone, Southern Ethiopia. BMC Public Health [Internet]. 2016;16:1240. Available from: 10.1186/s12889-016-3904-1 10.1186/s12889-016-3904-1 PMC5148861 27938363

[7] Campbell S (2006). Increasing immunisation coverage in developing countries. Prim Heal Care.

[8] Gibson DG, Tamrat T, Mehl G. The State of Digital Interventions for Demand Generation. Glob Heal Sci Pract. 2018;6(Supp):49–60. 10.9745/GHSP-D-18-00165 PMC6203418 30305339

[9] Jacobson Vann J, Jacobson R, Coyne-Beasley T, Asafu-adjei J, Szilagyi P (2018). Patient reminder and recall interventions to improve immunization rates (review). Cochrane Database Syst Rev.

[10] Kolff CA, Scott VP, Stockwell MS (2018). The use of technology to promote vaccination : a social ecological model based framework. Hum Vaccines Immunother ISSN.

[11] Atkinson KM, Wilson K, Murphy MSQ, El-halabi S, Kahale LA, Laflamme LL (2019). Effectiveness of digital technologies at improving vaccine uptake and series completion – a systematic review and meta-analysis of randomized controlled trials. Vaccine..

[12] Eze GU, Okojie OH. What experts think about integrating mobile health into routine immunization service delivery in Nigeria. mHealth [Internet]. 2016;2(1):1–7. Available from: http://www.ncbi.nlm.nih.gov/pubmed/28293579%0Ahttp://www.pubmedcentral.nih.gov/articlerender.fcgi?artid=PMC5344116 10.3978/j.issn.2306-9740.2016.01.01 PMC5344116 28293579

[13] Yunusa U, Garba SN, Umar AB, Idris SH, Bello UL, Abdulrashid I (2021). Mobile phone reminders for enhancing uptake, completeness and timeliness of routine childhood immunization in low and middle income countries: a systematic review and meta-analysis. Vaccine..

[14] Eze P, Lawani LO, Acharya Y (2021). Short message service (SMS) reminders for childhood immunization in low- and middle-income countries: a systematic review and meta-analysis. BMJ Glob Heal..

[15] Mekonnen ZA, Gelaye KA, Were MC, Gashu KD. Effect of mobile text message reminders on routine childhood vaccination : a systematic review and meta-analysis 2019;1–14. 10.1186/s13643-019-1054-0 PMC6598255 31253186

[16] Oliver-Williams C, Brown E, Devereux S, Fairhead C, Holeman I (2017). Using mobile phones to improve vaccination uptake in 21 low-and middle-income countries: systematic review. JMIR mHealth uHealth.

[17] Perski O, Short CE. Acceptability of digital health interventions: embracing the complexity. Transl Behav Med. 2021;(May):1–8. 10.1093/tbm/ibab048 PMC8320880 33963864

[18] United Nations Children Fund (UNICEF) Nigeria. News note: 4.3 million children in Nigeria still miss out on vaccinations every year [Internet]. Press release. 2018 [cited 2021 Jan 22]. Available from: https://www.unicef.org/nigeria/press-releases/news-note-43-million-children-nigeria-still-miss-out-vaccinations-every-year

[19] World Health Organization. Immunization coverage [Internet]. Fact sheets. 2020 [cited 2021 Jun 12]. Available from: https://www.who.int/news-room/fact-sheets/detail/immunization-coverage

[20] Eze P, Agu UJ, Aniebo CL, Agu SA, Lawani LO, Acharya Y. Factors associated with incomplete immunization in children aged 12-23 months at sub-national level, Nigeria: a cross-sectional study. BMJ open [internet]. 2021;11:e047445. Available from. 10.1136/bmjopen-2020-047445. 10.1136/bmjopen-2020-047445 PMC8237740 34172548

[21] Aworabhi-Oki N, Numbere T, Balogun MS, Usman A, Utulu R, Ebere N (2020). Trends in measles cases in Bayelsa state, Nigeria: a five-year review of case-based surveillance data (2014-2018). BMC Public Health.

[22] Ibrahim BS, Usman R, Mohammed Y, Datti Z, Okunromade O, Abubakar AA (2019). Burden of measles in Nigeria: a five-year review of casebased surveillance data, 2012-2016. Pan Afr Med J.

[23] Adeloye D, Jacobs W, Amuta AO, Ogundipe O, Mosaku O, Gadanya MA (2017). Coverage and determinants of childhood immunization in Nigeria: a systematic review and meta-analysis. Vaccine [Internet].

[24] Yaguo Ide L (2020). E G-JN. Vaccination status of children in Umueble Community in Rivers State, southern Nigeria and factors influencing it. Innov J Med Heal Sci.

[25] Sato R. Differential determinants and reasons for the non- and partial vaccination of children among Nigerian caregivers. Vaccine [internet]. 2020;38(1):63–9. Available from. 10.1016/j.vaccine.2019.09.097. 10.1016/j.vaccine.2019.09.097 31615717

[26] Akwataghibe NN, Ogunsola EA, Broerse JEW, Popoola OA, Agbo AI, Dieleman MA (2019). Exploring Factors Influencing Immunization Utilization in Nigeria—A Mixed Methods Study. Front Public Heal.

[27] Adefolalu OA, Kanma-Okafor OJ, Balogun MR (2019). Maternal knowledge, attitude and compliance regarding immunization of under five children in primary health care centres in Ikorodu local government area, Lagos State. J Clin Sci.

[28] Obasohan PE, Mustapha MA, Makada A, Obasohan DN (2018). Evaluating the reasons for partial and non-immunization of children in Wushishi local government area, Niger state, Nigeria: methodological comparison. Afr J Reprod Health.

[29] Forenbacher I, Husnjak S, Cvitić I, Jovović I. Determinants of mobile phone ownership in Nigeria. Telecomm policy [internet]. 2019;43(7):101812. Available from: 10.1016/j.telpol.2019.03.001.

[30] Vota W. The Main Drivers of Mobile Phone Ownership Will Surprise You [Internet]. ICTworks. 2019 [cited 2021 Jan 22]. Available from: https://www.ictworks.org/main-drivers-mobile-phone-ownership/#.YAzwCuhKjIW

[31] Kawakatsu Y, Oyeniyi Adesina A, Kadoi N, Aiga H (2020). Cost-effectiveness of SMS appointment reminders in increasing vaccination uptake in Lagos, Nigeria: a multi-centered randomized controlled trial. Vaccine..

[32] Ekhaguere OA, Oluwafemi RO, Badejoko B, Oyeneyin LO, Butali A, Lowenthal ED (2019). Automated phone call and text reminders for childhood immunisations (PRIMM): a randomised controlled trial in Nigeria. BMJ Glob Heal.

[33] Brown VB, Oluwatosin OA, Akinyemi JO, Adeyemo AA (2016). Effects of community health nurse-led intervention on childhood routine immunization completion in primary health care centers in Ibadan. Nigeria J Community Health.

[34] Stroup DF, Berlin JA, Morton SC, Olkin I, Williamson GDRD (2000). MOOSE guidelines for Meta-analyses and systematic reviews of observational studies. JAMA..

[35] Moher D, Liberati A, Tetzlaff J, Altman DG, Altman D, Antes G, et al. Preferred reporting items for systematic reviews and meta-analyses: the PRISMA statement. Ann Intern Med. 2009. PMC3090117 21603045

[36] Wells G, Shea B, O’Connell D, Peterson J (2000). The Newcastle-Ottawa scale (NOS) for assessing the quality of nonrandomised studies in meta-analyses.

[37] Tufanaru C, Munn Z, Aromataris E, Campbell J, Hopp L. Systematic reviews of effectiveness. In: Aromataris E, Munn Z, editors. JBI Manual for Evidence Synthesis [Internet]. 2020. Available from: https://synthesismanual.jbi.global

[38] Munn Z, Moola S, Lisy K, Riitano D, Tufanaru C. Systematic reviews of prevalence and incidence. In: Aromataris E, Munn Z, editors. JBI Manual for Evidence Synthesis [Internet]. 2020. Available from: https://synthesismanual.jbi.global.

[39] Munn Z, MClinSc SM, Lisy K, Riitano D, Tufanaru C. (2015). Methodological guidance for systematic reviews of observational epidemiological studies reporting prevalence and cumulative incidence data. Int J Evid Based Healthc.

[40] Nyaga VN, Arbyn M, Aerts M (2014). METAPROP: Stata module to perform fixed and random effects meta-analysis of proportions [internet].

[41] Nyaga VN, Arbyn M, Aerts M (2014). Metaprop: a Stata command to perform meta-analysis of binomial data. Arch Public Heal.

[42] Hunter JP, Saratzis A, Sutton AJ, Boucher RH, Sayers RD, Bown MJ (2014). In meta-analyses of proportion studies, funnel plots were found to be an inaccurate method of assessing publication bias. J Clin Epidemiol [Internet].

[43] Guyatt G, Oxman AD, Akl EA, Kunz R, Vist G, Brozek J (2011). GRADE guidelines: 1. Introduction - GRADE evidence profiles and summary of findings tables. J Clin Epidemiol.

[44] Ajayi AG, Balogun MR, Oladele DA (2015). Knowledge and attitudes towards mobile phone use to promote maternal and child health among women in Mushin, Lagos state. UNILAG J Med Sci Technol.

[45] Akinrinade OT, Ajayi IO, Fatiregun AA, Isere EE, Yusuf BO (2018). Ownership of mobile phones and willingness to receive childhood immunisation reminder messages among caregivers of infants in Ondo state, South-Western Nigeria. SAJCH South African J Child Heal.

[46] Ibraheem RM, Akintola MA, Abdulkadir MB, Adeboye MAN, Mohammed MJ (2018). A comparative analysis of mother’ preference for specific of phone-derived reminders for routine immunization appointments in Ilorin. Nigeria J Med Trop.

[47] Odinaka KK, Edelu BO, Achigbu KI (2018). Acceptance of mobile phone short message service for childhood immunization reminders by Nigerian mothers. Port Harcourt Med J.

[48] Oladepo O, Dipeolu IO, Oladunni O. Nigerian rural mothers’ knowledge of routine childhood immunizations and attitudes about use of reminder text messages for promoting timely completion. J public health policy [internet]. 2019;40(4):459–77. Available from. 10.1057/s41271-019-00180-7. 10.1057/s41271-019-00180-7 PMC7771534 31427672

[49] Onoja-Alexander M, Isa AS, Lawal B, Olorukooba A, Gobir A, Nwankwo B, et al. Awareness, Perception and Preferences of Mothers towards Mobile Phone Reminders for Routine Childhood Immunization Appointments in Ahmadu Bello University Teaching Hospital Shika,Zaria,Nigeria. Int J Infect Dis [Internet]. 2018;73S(2018):113. Available from: 10.1016/j.ijid.2018.04.3674

[50] Sadoh AE, Okungbowa E (2014). Nigerian mothers opinion of reminder/recall for immunization. Niger J Paediatr.

[51] Tagbo BN, Okafor V, Okolie S (2020). Acceptability of the use of reminder/recall in vaccination services among clients and service providers in Enugu. Nigeria Vaccines Vaccin Open Access.

[52] Balogun MR, Sekoni AO, Okafor IP, Odukoya OO, Ezeiru SS, Ogunnowo BE (2012). Access to information technology and willingness to receive text message reminders for childhood immunisation among mothers attending a tertiary facility in Lagos, Nigeria. SAJCH South African J Child Heal..

[53] Bello SO, Ruben JC, Ibrahim OR, Issa H, Ogunkunle TO, Afolabi OF (2020). The role of short message service (SMS) on childhood immunization in a tertiary health facility in Nasarawa state. Ann Clin Biomed Res.

[54] Brown VB, Oluwatosin OA (2017). Feasibility of implementing a cellphone-based reminder/recall strategy to improve childhood routine immunization in a low-resource setting: a descriptive report. BMC Health Serv Res.

[55] Brown VB, Oluwatosin A, Ogundeji MO (2015). Experiences, perceptions and preferences of mothers towards childhood immunization reminder/recall in Ibadan. Nigeria: a cross-sectional study Pan Afr Med J.

[56] Dipeolu IO (2017). Effect of Mobile-phone reminder text messages on mothers’ knowledge and completion of routine immunization in rural areas of Oyo state.

[57] Eze GU, Adeleye OO (2015). Enhancing routine immunization performance using innovative Technology in an Urban Area of Nigeria. West Afr J Med.

[58] Eze NC, Azuogu BN, Nwonwu EU, Agu AP (2018). Awareness, perception to immunisation reminders and recall among caregivers of infants in Abakaliki, Southeast Nigeria. Asian J Adv Res Reports.

[59] Ibraheem RM, Akintola MA (2017). Acceptability of reminders for immunization appointments via mobile devices by mothers in Ilorin, Nigeria: a cross-sectional study. Oman Med J.

[60] Eze NC, Onwasigwe CN (2018). Effect of Mobile phone reminders and recalls on Pentavalent vaccines drop-out rate among caregivers accessing childhood immunisation Services in a Developing City, Southeast Nigeria. Asian J Res Infect Dis.

[61] Eze NC, Azuogu BN, Okoronkwo IL (2018). Effect of Mobile phone reminders and recalls on missed Immunisations among infants in two health facilities in Abakaliki. Nigeria Asian J Pediatr Res.

[62] Oladepo O, Dipeolu IO, Oladunni O. Outcome of reminder text messages intervention on completion of routine immunization in rural areas. Nigeria Health Promot Int. 2020:1–9. 10.1093/heapro/daaa092 PMC8384379 33057615

[63] Ozawa S, Zhou M, Wonodi C, Chen HH, Bridges JFP. Parents’ preferences for interventions to improve childhood immunization uptake in northern Nigeria. Vaccine [internet]. 2018;36(20):2833–41. Available from. 10.1016/j.vaccine.2018.03.073. 10.1016/j.vaccine.2018.03.073 29661582

[64] Adedokun A, Idris O, Odujoko T (2016). Patients’ willingness to utilize a SMS-based appointment scheduling system at a family practice unit in a developing country. Prim Heal Care Res Dev.

[65] Emuoyibofarhe ON, Meinel C, Famutimi RF, Famutimi T, Anja P (2015). A repository Mobile immunization reminder system (RMIRS) for nursing mothers. Int J Comput.

[66] Brown VB (2014). Effects of community-based nursing intervention on routine childhood immunization completion in Ibadan Oyo state [internet].

[67] Eze NC, Onwasigwe CN, Una AF (2018). Implementation of Mobile phone reminder system to improve immunisation uptake in Abakaliki, southeast, Nigeria: its feasibility and acceptability. Asian J Med Princ Clin Pract.

[68] Jennings L, Omoni A, Akerele A, Ibrahim Y, Ekanem E (2015). Disparities in mobile phone access and maternal health service utilization in Nigeria: a population-based survey. Int J Med Inform [Internet].

[69] Dearing JW (2009). Applying diffusion of innovation theory to intervention development. Res Soc Work Pract.

[70] Olamoyegun MA, Emuoyibofarhe OJ, Ala OA, Ugwu E (2020). Mobile phone use in the Management of Diabetes in Nigeria: a new potential weapon. West Afr J Med.

[71] Dasuki SI, Zamani ED (2019). Assessing mobile phone use by pregnant women in Nigeria: a capability perspective. Electron J Inf Syst Dev Ctries.

[72] Ophori EA, Tula MY, Azih AV, Okojie R, Ikpo EP (2014). Current trends of immunization in Nigeria: Prospect and challenges. Trop Med Health.

[73] National Population Commission (NPC) Nigeria, ICF. Nigeria Demographic Health Survey 2018 [Internet]. Abuja, Nigeria and Rockville, Maryland, USA; 2019. Available from: https://dhsprogram.com/publications/publication-fr359-dhs-final-reports.cfm

