# Supplementary material for: Acceptability of mobile-phone reminders for routine childhood vaccination appointments in Nigeria – a systematic review and meta-analysis
Source: BMC Health Serv Res. 2021 Nov 26;21:1276. doi: 10.1186/s12913-021-07296-1 (PMC8627092; doi:10.1186/s12913-021-07296-1)
Supplement: Supplementary file 1 — Additional file 1: Supplement 1. Search strategy [file 12913_2021_7296_MOESM1_ESM.docx]

**Supplement 1:** Search strategy and terms

| **Database** | **Results** |
| --- | --- |
| **PubMed / MEDLINE**: <https://pubmed.ncbi.nlm.nih.gov/> |  |
| ((((("mobile"[All Fields] OR "mobiles"[All Fields]) AND "phone*"[All Fields]) OR ("text messaging"[MeSH Terms] OR ("text"[All Fields] AND "messaging"[All Fields]) OR "text messaging"[All Fields] OR ("short"[All Fields] AND "message"[All Fields] AND "service"[All Fields]) OR "short message service"[All Fields]) OR ("text messaging"[MeSH Terms] OR ("text"[All Fields] AND "messaging"[All Fields]) OR "text messaging"[All Fields] OR ("sms"[All Fields] AND "text"[All Fields]) OR "sms text"[All Fields]) OR ("text messaging"[MeSH Terms] OR ("text"[All Fields] AND "messaging"[All Fields]) OR "text messaging"[All Fields] OR ("text"[All Fields] AND "message"[All Fields]) OR "text message"[All Fields]) OR (("phone s"[All Fields] OR "phoned"[All Fields] OR "phones"[All Fields] OR "phoning"[All Fields] OR "telephone"[MeSH Terms] OR "telephone"[All Fields] OR "phone"[All Fields]) AND "call*"[All Fields])) AND ("remind"[All Fields] OR "reminded"[All Fields] OR "reminder"[All Fields] OR "reminders"[All Fields] OR "reminding"[All Fields] OR "remindings"[All Fields] OR "reminds"[All Fields] OR ("reminder systems"[MeSH Terms] OR ("reminder"[All Fields] AND "systems"[All Fields]) OR "reminder systems"[All Fields] OR ("reminder"[All Fields] AND "system"[All Fields]) OR "reminder system"[All Fields]))) OR ((("smart mater struct"[Journal] OR "sms"[All Fields]) AND ("remind"[All Fields] OR "reminded"[All Fields] OR "reminder"[All Fields] OR "reminders"[All Fields] OR "reminding"[All Fields] OR "remindings"[All Fields] OR "reminds"[All Fields])) OR (("text messaging"[MeSH Terms] OR ("text"[All Fields] AND "messaging"[All Fields]) OR "text messaging"[All Fields] OR ("text"[All Fields] AND "message"[All Fields]) OR "text message"[All Fields]) AND ("remind"[All Fields] OR "reminded"[All Fields] OR "reminder"[All Fields] OR "reminders"[All Fields] OR "reminding"[All Fields] OR "remindings"[All Fields] OR "reminds"[All Fields])) OR "phone call reminder"[All Fields])) AND ((("childhood"[All Fields] OR "childhoods"[All Fields]) AND ("immune"[All Fields] OR "immuned"[All Fields] OR "immunes"[All Fields] OR "immunisation"[All Fields] OR "vaccination"[MeSH Terms] OR "vaccination"[All Fields] OR "immunization"[All Fields] OR "immunization"[MeSH Terms] OR "immunisations"[All Fields] OR "immunizations"[All Fields] OR "immunise"[All Fields] OR "immunised"[All Fields] OR "immuniser"[All Fields] OR "immunisers"[All Fields] OR "immunising"[All Fields] OR "immunities"[All Fields] OR "immunity"[MeSH Terms] OR "immunity"[All Fields] OR "immunization s"[All Fields] OR "immunize"[All Fields] OR "immunized"[All Fields] OR "immunizer"[All Fields] OR "immunizers"[All Fields] OR "immunizes"[All Fields] OR "immunizing"[All Fields])) OR (("childhood"[All Fields] OR "childhoods"[All Fields]) AND ("vaccin"[Supplementary Concept] OR "vaccin"[All Fields] OR "vaccination"[MeSH Terms] OR "vaccination"[All Fields] OR "vaccinable"[All Fields] OR "vaccinal"[All Fields] OR "vaccinate"[All Fields] OR "vaccinated"[All Fields] OR "vaccinates"[All Fields] OR "vaccinating"[All Fields] OR "vaccinations"[All Fields] OR "vaccination s"[All Fields] OR "vaccinator"[All Fields] OR "vaccinators"[All Fields] OR "vaccine s"[All Fields] OR "vaccined"[All Fields] OR "vaccines"[MeSH Terms] OR "vaccines"[All Fields] OR "vaccine"[All Fields] OR "vaccins"[All Fields])) OR (("routine"[All Fields] OR "routinely"[All Fields] OR "routines"[All Fields] OR "routinization"[All Fields] OR "routinize"[All Fields] OR "routinized"[All Fields] OR "routinizing"[All Fields]) AND ("immune"[All Fields] OR "immuned"[All Fields] OR "immunes"[All Fields] OR "immunisation"[All Fields] OR "vaccination"[MeSH Terms] OR "vaccination"[All Fields] OR "immunization"[All Fields] OR "immunization"[MeSH Terms] OR "immunisations"[All Fields] OR "immunizations"[All Fields] OR "immunise"[All Fields] OR "immunised"[All Fields] OR "immuniser"[All Fields] OR "immunisers"[All Fields] OR "immunising"[All Fields] OR "immunities"[All Fields] OR "immunity"[MeSH Terms] OR "immunity"[All Fields] OR "immunization s"[All Fields] OR "immunize"[All Fields] OR "immunized"[All Fields] OR "immunizer"[All Fields] OR "immunizers"[All Fields] OR "immunizes"[All Fields] OR "immunizing"[All Fields])) OR 19[UID]) AND ("accept"[All Fields] OR "acceptabilities"[All Fields] OR "acceptability"[All Fields] OR "acceptable"[All Fields] OR "acceptably"[All Fields] OR "acceptance"[All Fields] OR "acceptances"[All Fields] OR "acceptation"[All Fields] OR "accepted"[All Fields] OR "accepter"[All Fields] OR "accepters"[All Fields] OR "accepting"[All Fields] OR "accepts"[All Fields] OR "willingness to receive"[All Fields] OR 23[UID] OR 24[UID]) AND ("nigeria"[MeSH Terms] OR "nigeria"[All Fields] OR "nigeria s"[All Fields]) | 3 |
|  |  |
| **AJOL:** <https://www.ajol.info/index.php/ajol> |  |
| mobile phone reminder* AND Nigeria | 128 |
|  |  |
| **CINAHL:** <http://web.b.ebscohost.com.ezaccess.libraries.psu.edu/ehost/search/advanced?vid=0&sid=ca3eb941-9c0f-41ee-9240-479d30e2b85f%40sessionmgr103> |  |
| mobile phone reminders AND Nigeria | 5 |
|  |  |
| **Cochrane Library:** [https://www-cochranelibrary-com.ezaccess.libraries.psu.edu/#](https://www-cochranelibrary-com.ezaccess.libraries.psu.edu/) |  |
| ((mobile phone reminders) AND Nigeria) in Title Abstract Keyword | 7 |
|  |  |
| **CNKI:** <https://global.cnki.net/index/> |  |
| “mobile phone reminder* AND Nigeria” in full text | 84 |
|  |  |
| **PsycINFO:** <https://www-proquest-com.ezaccess.libraries.psu.edu/psycinfo/advanced?accountid=13158> |  |
| ab(reminders) AND ab(vaccination) AND ab(Nigeria) | 1 |
|  |  |
| **Scopus:** <https://www-scopus-com.ezaccess.libraries.psu.edu/search/form.uri?display=basic#basic> |  |
| ( TITLE-ABS-KEY ( mobile AND phone AND reminders ) AND TITLE-ABS-KEY ( Nigeria ) ) | 17 |
|  |  |
| **Web of Science:** <https://apps-webofknowledge-com.ezaccess.libraries.psu.edu/UA_GeneralSearch_input.do?product=UA&SID=6C6n1joOGmcqKQvAtSB&search_mode=GeneralSearch> |  |
| (((ALL=(mobile-phone )) OR ALL=("mobile phone")) AND ALL=(reminders )) AND ALL=(Nigeria ) | 11 |
|  |  |
| *Databases available on Web of Science via institution* |  |
| 1. Arts & Humanities Citation Index (A&HCI)--1975-present; |  |
| 1. Book Citation Index – Science (BKCI-S)--2005-present |  |
| 1. Book Citation Index – Social Sciences & Humanities (BKCI-SSH)--2005-present |  |
| 1. Conference Proceedings Citation Index – Science (CPCI-S)--1990-present |  |
| 1. Conference Proceedings Citation Index – Social Science & Humanities (CPCI-SSH)--1990-present |  |
| 1. Current Chemical Reactions (CCR-EXPANDED)--1985-present |  |
| 1. Emerging Sources Citation Index (ESCI)--2015-present |  |
| 1. Index Chemicus (IC)--1993-present |  |
| 1. Science Citation Index Expanded (SCI-EXPANDED)--1900-present |  |
| 1. Social Sciences Citation Index (SSCI)--1900-present |  |
| 1. Arts & Humanities Citation Index (A&HCI)--1975-present; |  |
| 1. Book Citation Index – Science (BKCI-S)--2005-present |  |
|  |  |
| **TOTAL SEARCH RESULT** | **256** |
