# Supplementary figures and images for: Acceptability of mobile-phone reminders for routine childhood vaccination appointments in Nigeria – a systematic review and meta-analysis
Source: BMC Health Serv Res. 2021 Nov 26;21:1276. doi: 10.1186/s12913-021-07296-1 (PMC8627092; doi:10.1186/s12913-021-07296-1)

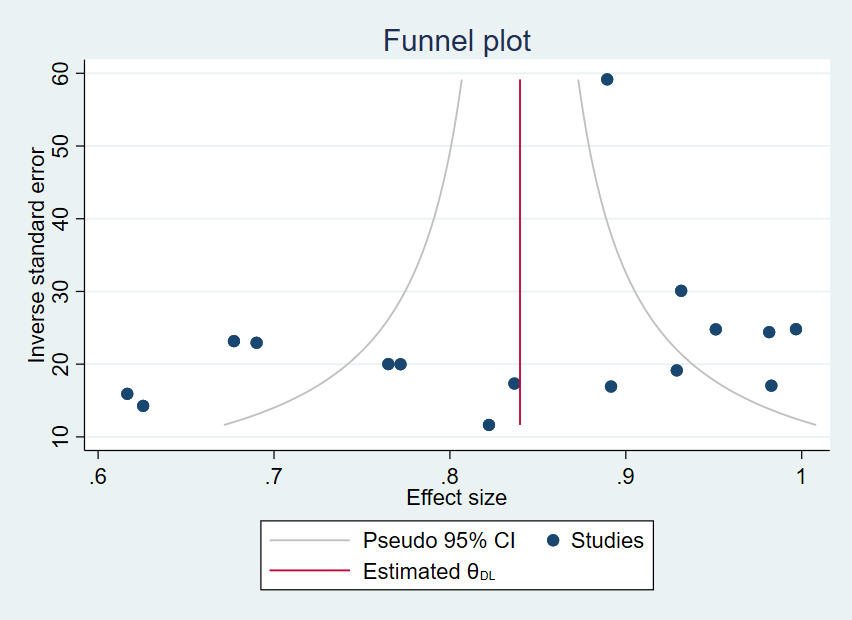

Supplement: Supplementary file 2 — Additional file 2: Supplement 2. Funnel plot for graphic assessment of publication bias [file 12913_2021_7296_MOESM2_ESM.png]

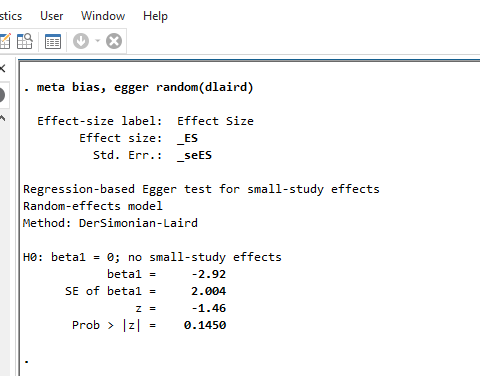

Supplement: Supplementary file 3 — Additional file 3: Supplement 3. Egger test for objective assessment for evidence of publication bias [file 12913_2021_7296_MOESM3_ESM.png]

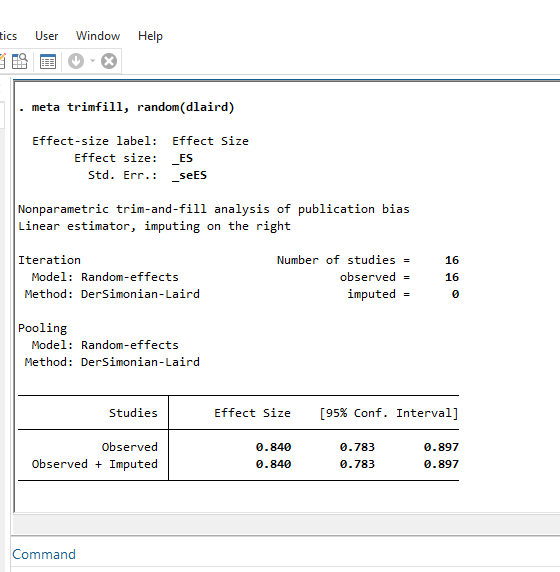

Supplement: Supplementary file 4 — Additional file 4: Supplement 4. Trim-and-fill method for estimating potentially missing studies due to publication bias [file 12913_2021_7296_MOESM4_ESM.png]
